# Chemical characteristics of wintertime PM_2.5_ in background region of northwest China during urban emission reduction

**DOI:** 10.1016/j.isci.2025.113528

**Published:** 2025-09-09

**Authors:** Yali Liu, Xiao Guo, Yifan Zhang, Yue Cao, Yingkun Jiang, Weining Qi, Minxia Shen, Lu Li, Qian Wang, Wenting Dai, Jianjun Li

**Affiliations:** 1 State Key Laboratory of Loess Science, Institute of Earth Environment, Chinese Academy of Sciences, Xi’an 710061, China; 2 Xi’an Institute for Innovative Earth Environment Research, Xi’an 710061, China; 3 Shaanxi Key Laboratory of Atmospheric and Haze-fog Pollution Prevention, Xi’an 710061, China; 4 National Observation and Research Station of Regional Ecological Environment Change and Comprehensive Management in the Guanzhong Plain, Xi’an Shaanxi, China

**Keywords:** Natural sciences, Environmental science, Environmental monitoring

## Abstract

To investigate the impact of urban emission reduction on particulate matter (PM_2.5_) pollution characteristics, field observations were conducted near a megacity in the Guanzhong Plain, China, during three periods: COVID-19 lockdown (“lockdown”), pre-lockdown (“normal”), and post-lockdown (“festival”). The observation showed that despite reduced NO_2_ and SO_2_, PM_2.5_ increased significantly during “lockdown.” Molecular characteristics and PMF source apportionment revealed that biomass burning contributions to PM_2.5_ increased by over 70% compared with “normal.” Meanwhile, secondary aerosol formation (primarily through liquid-phase oxidation) accounted for more than 50% of PM_2.5_ levels during “lockdown.” Additionally, metal ions released by fireworks burning accelerated the liquid-phase formation of sulfate, resulting in secondary sulfate-related sources contributing about 33% of PM_2.5_ during “festival.” The study demonstrates synergistic effects between biomass/fireworks burning and liquid-phase oxidation, indicating that unbalanced emission reductions may exacerbate pollution through atmospheric aging and regional transport. Effective air quality management requires coordinated multi-pollutant control strategies.

## Highlights

• The contribution of biomass burning to PM_2.5_ increased by over 70% during lockdown
• Secondary aerosol formation accounted for more than 50% of PM_2.5_ levels in lockdown
• Liquid-phase oxidation was the primary way of secondary aerosol formation
• Increased fireworks burning could accelerate the sulfate formation during festival

## Graphical abstract

**Figure:**
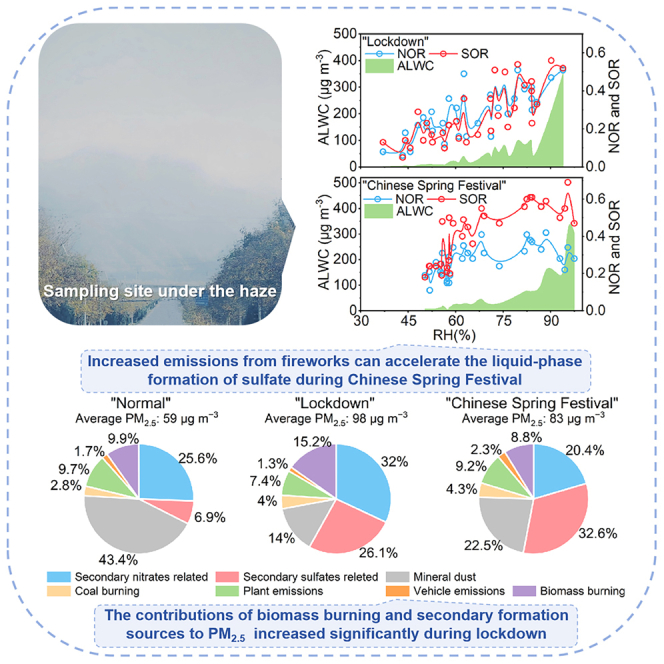

## Introduction

Particulate matter (PM_2.5_) adversely impacts regional climate and human health, being emitted primarily from natural or anthropogenic sources and also being formed secondarily via gas-phase or liquid-phase oxidation.^1^^,^^2^ Rapid industrialization, urbanization, and transportation have exacerbated PM_2.5_ pollution in China over the past few decades.^3^^,^^4^ In 2013, the Chinese State Council introduced the “Atmospheric Pollution Prevention and Control Action Plan,” which laid out a series of stringent emission reduction strategies aimed at controlling pollution from vehicles, industries, biomass/coal burning, and cooking. Although these measures have reduced anthropogenic pollutant precursors and somewhat alleviated PM_2.5_ pollution, severe haze persists in northwestern China, driven by the synergistic effects of pollutant accumulation, unfavorable meteorology, and topography.^5^^,^^6^^,^^7^ Recent studies have indicated that secondary aerosol formation significantly contributes to extreme haze events, in addition to primary emissions.

At the beginning of 2020, to control the spread of the 2019 novel coronavirus (COVID-19), rapid reductions in human activities worldwide were implemented, with measures such as imposing traffic restrictions, suspending factory operations, and enforcing home quarantines. These restrictions led to dramatic decreases in primary pollutant emissions such as CO, NOx, and anthropogenic volatile organic compounds, providing a rare opportunity to study the contribution of secondary aerosols to PM_2.5_ pollution.^8^^,^^9^ Some research has found that contributions from secondary sulfate and organic aerosols have increased, effectively offsetting reductions in vehicle-related and other primary emissions in urban locations during lockdown.^10^ Studies also confirmed that reduced NOx emissions from transportation increased O_3_ and nighttime NO_3_ radical formation, thereby enhancing atmospheric oxidation capacity and facilitating the formation of secondary PM_2.5_ during the lockdown period, which may lead to an increase in PM_2.5_ concentration instead of a decrease.^11^ However, many current studies predominantly employ satellite data and regional modeling to assess the impacts of urban emission reduction and largely overlook the changes in the molecular composition of PM_2.5_.^12^^,^^13^ Some studies utilize an aerodyne aerosol mass spectrometer (AMS) or aerosol chemical speciation monitor (ACSM) to provide real-time aerosol characterization and identify sources of PM_2.5_.^14^^,^^15^ Nonetheless, the specificity of AMS and ACSM for individual mass fragment ions is not as effective as using molecular markers for tracking and identifying sources. There is a notable lack of comprehensive molecular-level research on PM_2.5_, particularly in terms of detailed studies on the molecular composition of organic aerosols, which results in unclear mechanisms behind the increase in secondary PM_2.5_ at the molecular level following reductions in anthropogenic emissions.

Many studies on the atmospheric effects of the COVID-19 lockdown have primarily focused on urban environments characterized by high traffic and industrial emissions. The emission intensity in suburban areas is significantly lower than that in urban settings, and it is considerably influenced by the emissions from nearby urban areas. The complex aging process that occurs during aerosol transmission can lead to distinct pollution characteristics between suburban and urban environments, resulting in different responses to pollution control measures for PM_2.5_. Thus, field observation data at the molecular level in suburban areas are essential to accurately assess the impact of reducing urban pollution sources on the composition of PM_2.5_ in surrounding regions. The Guanzhong Plain, as the economic hub of northwest China, faces persistent air quality challenges due to intensive industrialization and urban expansion.^16^^,^^17^ Geographically, it is constrained by the Loess Plateau to the north and the Qinling Mountains to the south, with only an opening to the east. Unfavorable terrain accumulates and circulates pollutants within the plain, worsening PM_2.5_ pollution, notably through secondary aerosol formation. Xi’an is a core megacity in the Guanzhong Plain, which generates significant anthropogenic emissions and significantly impacts the air quality in surrounding areas. To prevent the spread of COVID-19, Xi’an implemented citywide closure and control measures from December 23, 2021–January 23, 2022. During this period, travel for traffic, industry, and residents was strictly restricted, creating an excellent opportunity to assess the effects of anthropogenic pollution emission control in a megacity on the atmospheric environment of suburban regions. The northern foothills of the Qinling Mountains, situated in the southern part of the Guanzhong Plain and with minimal local pollution sources, served as an ideal background site for evaluating the regional impact of megacity pollution emission controls.

This study focuses on a background point at the northern foot of the Qinling Mountains as the research area, investigating the influence of emission reductions during the Xi’an lockdown on PM_2.5_ composition and the underlying contributing sources. The measurement campaign spanned the pre-lockdown phase (November 24–December 22, 2021) and the lockdown phase (December 23, 2021–January 23, 2022). In addition, the lifting of the lockdown in Xi’an coincided with the Chinese Spring Festival period (January 24–February 24, 2022), allowing for further assessment of the impacts of fireworks burning on regional air quality. Detailed measurements of the primary chemical composition of PM_2.5_ include water-soluble inorganic ions (WSIIs), organic carbon (OC), elemental carbon (EC), water-soluble organic carbon (WSOC), and organic tracer compounds. The goals are to quantify the contribution of potential sources and elucidate atmospheric response in background regions to megacity emission changes through comparative characterization of PM_2.5_ chemical composition across three typical periods. Direct field observation evidence can reflect the crucial impact of urban emission reduction on regional air quality and provide scientific guidance for refining future air pollution control strategies.

## Results and discussion

### Overall trend of the leading observational indicators

**Figure 1:**
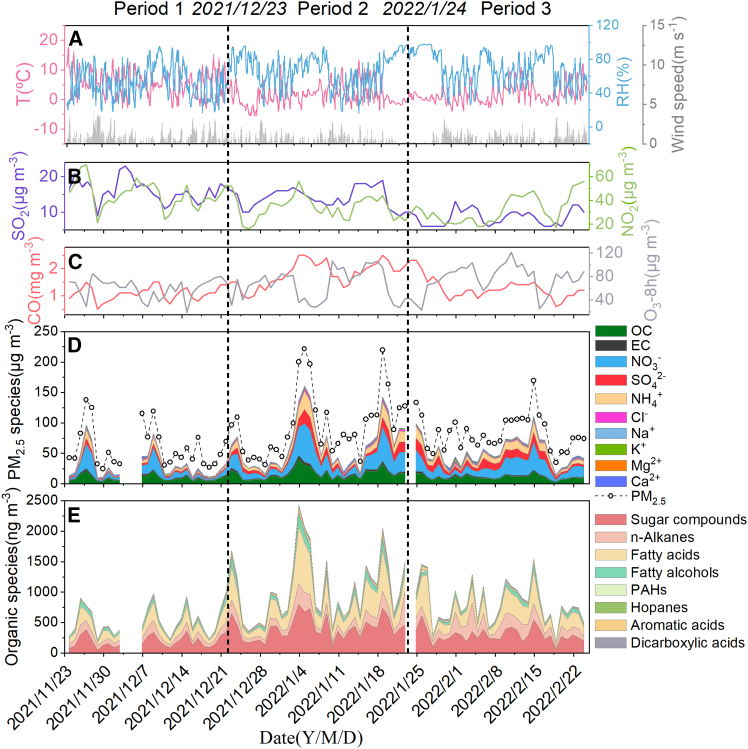
Temporal variation of the main observed indicators Temporal variation in meteorological parameters (A) and concentrations of major chemical compositions (B–E) in the whole observation period.

Temporal variations in the concentrations of gaseous pollutants (SO_2_, NO_2_, CO, and O_3_-8h), PM_2.5_, OC, EC, WSOC, WSIIs, and organic compounds, along with meteorological parameters (temperature T, relative humidity [RH], and wind speed) in the whole observation period, are presented in Figure 1. Based on human activities and special events, we divided the observation period into three: Period 1 (P1), from November 24–December 22, 2021, when industrial production and daily life continued as usual, was defined as “normal”; Period 2 (P2), from December 23, 2021–January 23, 2022, when Xi’an implemented citywide closure and control measures to curb the spread of COVID-19, was defined as “lockdown”; Period 3 (P3), from January 24–February 24, 2022, when the lifting of the lockdown in Xi’an coincided with the Chinese Spring Festival period and fireworks burning increased significantly compared with other periods, was defined as “festival.”

**Figure 2:**
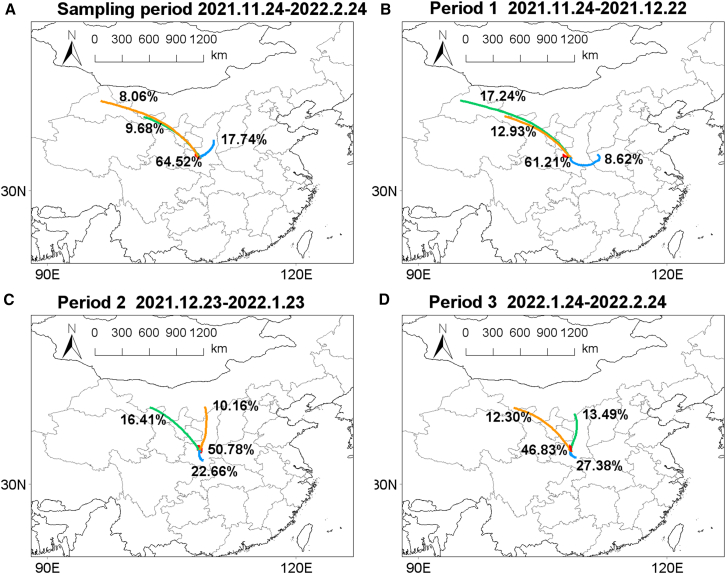
48-hour air mass backward trajectories Air mass backward trajectories throughout the sampling period (A), during Period 1 (“normal,” November 24–December 22, 2021) (B), Period 2 (“lockdown,” December 23, 2021–January 23, 2022) (C), and Period 3 (Chinese Spring Festival: “festival,” January 24–Feb 24, 2022) (D).

During the entire sampling period, PM_2.5_ concentrations ranged from 24.64 to 221.74 μg m^−3^, with a mean value of 81.4 ± 42.96 μg m^−3^, lower than previous reports on urban areas in the Guanzhong Plain,^18^^,^^19^ which may be attributed to the fact that the sampling site is located at the edge of the Qinling Mountains, far away from residential areas, so there are fewer local emission sources. However, the region still experienced several severe pollution processes with PM_2.5_ concentrations surpassing 150 μg m^−3^, likely due to pollution transport from urban areas. During P1, atmospheric conditions are relatively good, the RH and wind speed are 53.98% ± 13.31% and 0.74 ± 0.55 m s^−1^, respectively, with an average concentration of PM_2.5_ of 59.36 ± 33.03 μg m^−3^. Compared with P1, the concentration of NO_2_ in P2 decreased significantly (44.04 ± 11.79 μg m^−3^ in P1 and 34.06 ± 9.72 μg m^−3^ in P2), which should be due to traffic restrictions. However, the concentration of PM_2.5_ significantly increased during P2, with an average of 97.61 ± 53.70 μg m^−3^. Two heavy pollution event processes with PM_2.5_ exceeding 200 μg m^−3^ occurred in P2, which may be related to the deterioration of atmospheric conditions, marked by higher RH levels (65.93 ± 15.47%) and diminished wind speed (0.49 ± 0.36 m s^−1^). At the same time, the concentrations of inorganic ions and organic aerosols also increased significantly. During P3, the atmospheric conditions were still dominated by high RH (69.18% ± 15.09%) and low wind speed (0.58 ± 0.38 m s^−1^). The average PM_2.5_ concentration is relatively high at 83.26 ± 28.63 μg m^−3^, which is 1.4 times higher than that observed in P1. Backward trajectories of air masses indicate that the source of short-range air masses from surrounding areas is greater than 60%, suggesting that human pollution in these regions, such as Xi’an, significantly impacts the sampling point (Figure 2A). Moreover, the backward trajectories of air masses in the three stages are similar and the contributions of short-distance sources are all over 60% (Figures 2B–2D). Therefore, the differences in atmospheric pollution characteristics during these three periods may be jointly influenced by close-range emission sources and atmospheric aging processes.

### The major chemical compositions of PM_2.5_ in the three periods

**Figure 3:**
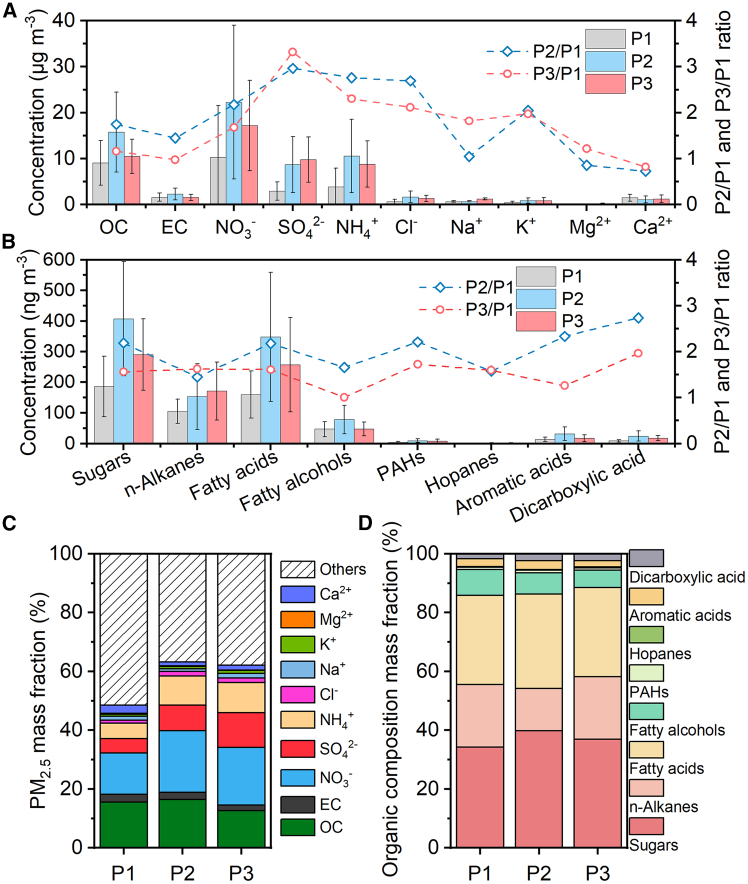
Mass concentration and percentage of major chemical species The concentrations of OC, EC, and WSIIs (A) and their concentration percentage in PM_2.5_ (C); concentrations of organic compounds (B) and their concentration percentage in total detected organic compounds (D). Error bars in (A) and (B) represent SDs of the averages from measurements.

The concentrations of carbonaceous components (OC, EC, and WSOC) and WSIIs (NO_3_^−^, SO_4_^2−^, Cl^−^, NH_4_^+^, Na^+^, K^+^, Mg^2+^, and Ca^2+^) in PM_2.5_ during the three stages throughout the sampling period are summarized in Figure 3A. Detailed average concentrations, concentration percentages, and diagnostic ratios of the major chemical compositions are summarized in Table S1. The total concentrations of the major chemical composition in PM_2.5_ samples detected are 30.97 ± 23.52 μg m^−3^, 64.02 ± 40.71 μg m^−3^, and 52.49 ± 22.45 μg m^−3^, in P1, P2, and P3, accounting for 48.54% ± 12.37%, 63.17% ± 11.04%, and 62.08% ± 11.43% of the PM_2.5_, respectively. The proportion of the major chemical compositions detected in PM_2.5_ during P2 and P3 was significantly higher than that in P1 (Figure 3C).

#### OC, EC, and WSOC

**Figure 4:**
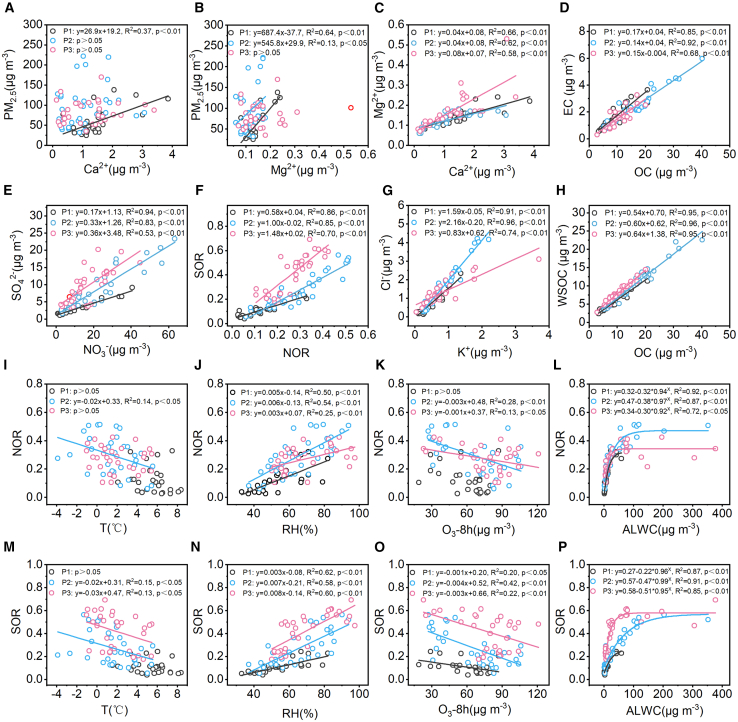
Cross-correlations between major chemical compositions of PM_2.5_ The relationships between PM_2.5_ and Ca^2+^ (A), and Mg^2+^ (B); Ca^2+^ and Mg^2+^ (C); OC and EC (D), and WSOC (H); NO_3_^-^ and SO_4_^2-^ (E); NOR and SOR (F); K^+^ and Cl^-^ (G); NOR and T (I), RH (J), O_3_-8h (K), and ALWC (L); SOR and T (M), RH (N), O_3_-8h (O), and ALWC (P).

The concentrations of OC are 9.04 ± 4.84 μg m^−3^, 15.77 ± 8.68 μg m^−3^, and 10.49 ± 3.72 μg m^−3^, in P1, P2, and P3, accounting for 15.55% ± 2.95% of PM_2.5_ in P1, slightly increasing to 16.46% ± 3.65% in P2 but significantly decreasing in P3 (12.63% ± 2.01%). The concentrations of EC are 1.59 ± 0.89 μg m^−3^, 2.30 ± 1.30 μg m^−3^, and 1.55 ± 0.66 μg m^−3^ in P1, P2, and P3, accounting for 2.71% ± 0.72%, 2.43% ± 0.75%, and 1.85% ± 0.55% of PM_2.5_, respectively. The average OC/EC ratios are 5.94 ± 1.15, 6.99 ± 1.07, and 7.25 ± 1.71, in P1, P2, and P3, respectively. The ratio of OC/EC at this background site is relatively higher than that in the Guanzhong Plain urban areas,^20^^,^^21^^,^^22^ which is reasonable, as fossil fuel burning typically results in a lower OC/EC ratio, whereas biomass burning generally produces a higher OC/EC ratio.^23^^,^^24^ The sampling point is located in the suburbs, where decentralized biomass burning for cooking/heating plays a relatively more important role than vehicular emissions. As shown in Figure 4D, the strong linear correlation between OC and EC also proves their similar emission sources. The OC/EC ratio is highest in P3, which may be related to the fireworks burning during this period, as the organic materials used in fireworks production evaporate significantly when ignited.^25^ In addition, the elevated OC/EC values can also result from increased secondary organic aerosol formation.^26^ The overall trend of WSOC and OC concentration is consistent, and there is an undeniable linear correlation with OC, indicating that their sources are consistent (Figure 4H). The proportion of WSOC in OC exceeded 60% in all three periods, reaching 80% in P3. The WSOC/OC ratio can indicate the degree of atmospheric aging, and the high WSOC/OC values seen during the entire sampling period suggest that the aerosols have undergone a complex atmospheric aging process, particularly in P3.

#### Water-soluble inorganic ions

The concentrations of WSIIs in PM_2.5_ are 20.34 ± 18.37 μg m^−3^, 45.95 ± 32.16 μg m^−3^, and 40.46 ± 18.95 μg m^−3^ during P1, P2, and P3, respectively, accounting for 30.28% ± 11.31% of PM_2.5_ in P1, which is significantly lower than the percentages in P2 (44.29% ± 11.54%) and P3 (47.60% ± 11.54%). The difference is primarily attributed to the lower percentages of secondary inorganic ions ([SNAs] including NO_3_^−^, SO_4_^2−^, and NH_4_^+^) in PM_2.5_ during P1 (24.21% ± 12.31%) compared with P2 (39.56% ± 12.27%) and P3 (41.70% ± 13.18%), suggesting that there may not be strong secondary formation in P1 relative to the other periods. Sulfate oxidative rate (SOR) and nitrate oxidative rate (NOR) can indicate the extent of secondary conversion reactions of SO_4_^2−^ and NO_3_^−^.^27^^,^^28^ Generally, higher values of SOR and NOR signify a stronger degree of secondary transformation. Conversely, when these values are <0.10, SO_4_^2−^ and NO_3_^−^ primarily originate from direct emissions.^29^

During P1, the SOR and NOR are 0.12 ± 0.09 and 0.11 ± 0.06, respectively, indicating that secondary nitrate and sulfate formation during this period is fragile and primary emissions may have dominated. The proportions of Ca^2+^ and Mg^2+^ in PM_2.5_ are 2.84% ± 1.10% and 0.28% ± 0.10%, significantly higher than those in P2 and P3. Ca^2+^ and Mg^2+^ are typical mineral dust ions, mainly from soil, construction, and traffic fugitive dust.^30^ The higher concentration percentages may result from intensive construction activities and road traffic in P1. An apparent linear correlation between Ca^2+^ and Mg^2+^ (R^2^ = 0.66) also suggests that they share similar emission sources. Additionally, the relationships of PM_2.5_ with Ca^2+^ (R^2^ = 0.37) and Mg^2+^ (R^2^ = 0.64) are much more significant than in other periods, further demonstrating the importance of mineral dust to PM_2.5_ in P1 (Figures 4A–4C).

During P2, the proportions of Ca^2+^ and Mg^2+^ in PM_2.5_ are 1.31% ± 0.97% and 0.16% ± 0.08%, respectively, which are significantly lower compared with P1, mainly due to the decrease in building activities and road traffic resulting from Xi’an’s lockdown measures. In addition, relatively high RH (65.93% ± 15.47%) and stable atmospheric conditions further contributed to these changes. In contrast, the concentration percentage of SNA has increased significantly, accounting for 39.56% ± 12.27% of the PM_2.5_ concentration. The SOR and NOR are 0.27 ± 0.15 and 0.30 ± 0.14, respectively, indicating a strong formation of secondary nitrate and sulfate during this period. As shown in Figures 4I–4P, both SOR and NOR show negative correlations with T and O_3_-8h, while exhibiting strong positive correlations with RH (R^2^ = 0.58 and 0.54) and aerosol liquid water content (ALWC) (R^2^ = 0.91 and 0.87), proving that the formation of secondary nitrate and sulfate mainly comes from liquid-phase oxidation rather than photochemical oxidation.^31^ K^+^ is commonly used as a biomass-burning ion tracer, and when it has a strong correlation with Cl^−^, it is generally believed that both ions originate from biomass burning.^32^ In P2, the concentrations of K^+^ and Cl^−^, as well as their proportions in PM_2.5_, increased significantly and exhibited a strong linear correlation (R^2^ = 0.96), reflecting a notable impact from biomass burning emissions. Strict restrictions on outdoor activity are the main reason for the increase in biomass-burning emissions, as extended time spent at home may have boosted biomass use for cooking and heating, especially in suburban regions.^33^

**Figure 5:**
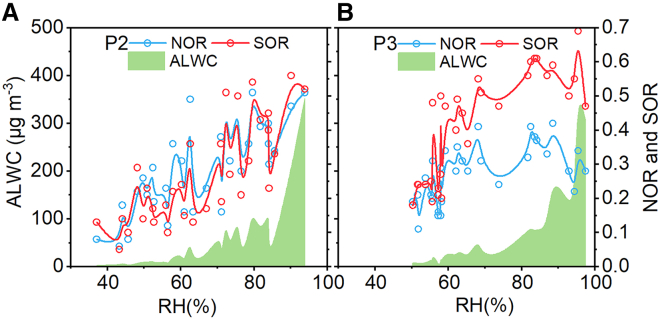
Dependence of NOR, SOR, and ALWC on RH The NOR, SOR, and ALWC trend increases with RH in P2 (A) and P3 (B).

During P3, the proportions of Ca^2+^ and Mg^2+^ in PM_2.5_ are 1.57% ± 1.07% and 0.22% ± 0.12%, respectively, which increased compared with P2 but remained much lower than in P1. On the one hand, the resumption of normal production and daily activities in Xi’an directly contributed to the rise in atmospheric concentrations of Ca^2+^ and Mg^2+^. On the other hand, persistent low wind speeds (0.58 ± 0.38 m s^−1^) may inhibit the resuspension of mineral dust, resulting in lower atmospheric concentrations of Ca^2+^ and Mg^2+^. Notably, the increase in Mg^2+^ concentration is more significant than that of Ca^2+^, and the correlation between Ca^2+^ and Mg^2+^ (R^2^ = 0.58) has weakened compared with other periods, which may be attributed to the fact that the sources of these two ions are not entirely the same during P3. Elevated concentrations of Mg^2+^ may be partially attributed to extensive emissions from fireworks in P3. Many previous studies have confirmed that Mg^2+^, along with SO_4_^2−^, Cl^−^, and K^+^, can all originate from fireworks burning.^25^ During this period, the proportions of SO_4_^2−^, Cl^−^, and K^+^ in PM_2.5_, the same as Mg^2+^, are the highest among the three periods, with values of 11.75% ± 4.78%, 1.54% ± 0.51%, and 0.96% ± 0.67%, respectively, confirming the substantial environmental impact of fireworks during that time. Under high RH conditions, the SOR and NOR are 0.42 ± 0.16 and 0.27 ± 0.09, respectively, showing negative correlations with T and O_3_-8h, while exhibiting positive correlations with RH (R^2^ = 0.60 and 0.25) and ALWC (R^2^ = 0.85 and 0.72), indicating that the formation of secondary nitrate and sulfate mainly comes from liquid-phase oxidation, similar to P2. It is interesting to note, as shown in Figure 5, that whereas the RHs of P3 (69.18% ± 15.09%) and P2 (65.93% ± 15.47%) are comparable, the SOR in P3 is significantly higher than NOR, unlike the essential consistency between SOR and NOR in P2, which may be due to the influence of fireworks burning in P3. This is because trace elements like K and Mg, as well as Fe and Cu, are widely used as fuels, oxidizers, and coloring agents in fireworks, which can be released into the atmosphere through the fireworks burning.^34^^,^^35^ Previous studies have demonstrated that metals (Fe, Cu, and others) play a significant catalytic role in the oxidation of SO_2_ in the liquid phase.^25^^,^^36^^,^^37^^,^^38^ The contribution of metal-catalyzed oxidation to SO_4_^2−^ can be as high as 49% ± 10% in the haze pollution.^39^ For Fe-catalyzed formation of SO_4_^2−^, the threshold of RH was around 85%, and for Cu-catalyzed reactions, the threshold of RH was 55%.^25^ The RH of P3 reached a maximum of 97.45%, providing favorable meteorological conditions for metal-catalyzed oxidation.

### Organic compound characteristics of PM_2.5_ in the three periods

More than 100 organic species were detected in PM_2.5_ samples, which were categorized into eight major classes according to their functional groups and emission sources, including sugar compounds, n-alkanes, fatty acids, fatty alcohols, polycyclic aromatic hydrocarbons (PAHs), hopanes, aromatic acids, and dicarboxylic acids. As detailed in Table S2, the total concentrations of these measured organics exhibited significant variability across sampling periods, averaging 527.07 ± 240.89 ng m^−3^ (P1), 1,054.84 ± 552.46 ng m^−3^ (P2), and 811.00 ± 340.77 ng m^−3^ (P3). Detailed average concentrations, concentration percentages, and diagnostic ratios of detected organic compounds are summarized in Figures 3B and 3D and Table S3.

#### Sugar compounds

**Figure 6:**
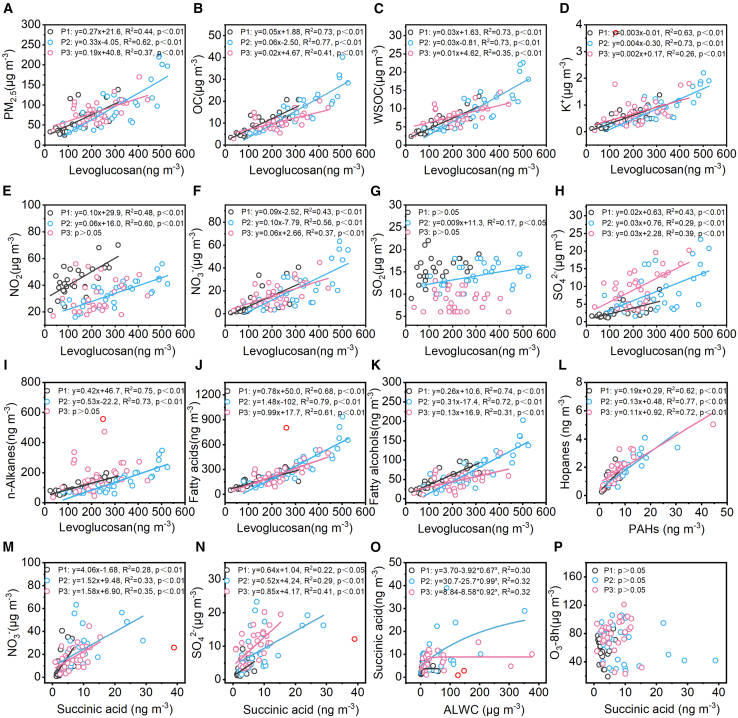
Cross-correlations between organic species of PM_2.5_ The relationships between levoglucosan and PM_2.5_ (A), OC (B), WSOC (C), K^+^ (D), NO_2_ (E), NO_3_^-^ (F), SO_2_ (G), SO_4_^2-^ (H), n-alkanes (I), fatty acids (J), and fatty alcohols (K); PAHs and hopanes (L); succinic acid and NO_3_^-^ (M), SO_4_^2-^ (N), ALWC (O), and O_3_-8h (P).

Sugar compounds consist of three anhydrosugars, four primary sugars, and three sugar alcohols. The total concentrations of anhydrosugars (levoglucosan, mannosan, and galactosan) are 165.39 ± 93.59 ng m^−3^, 377.35 ± 170.59 ng m^−3^, and 268.47 ± 109.78 ng m^−3^ in P1, P2, and P3, accounting for 29.95% ± 6.12%, 37.01% ± 5.84%, and 34.05% ± 7.96% of the total detected organic compounds, respectively. Anhydrosugars are primarily generated through the combustion and pyrolysis of cellulose and hemicellulose. Among them, levoglucosan has long been recognized as a key biomarker for biomass burning.^40^ However, recent studies have revealed that residential coal burning is also a significant source of atmospheric levoglucosan.^41^ The ratios between anhydrosugars are usually used to differentiate the types of burning substrates.^42^ The levoglucosan/mannosan (L/M) ratio between 0.23–33 and 31–189 denotes the biomass burning and lignite burning, respectively.^43^ The L/M ratio is 14.61 ± 4.43 in P1, 13.00 ± 4.21 in P2, and 16.79 ± 6.50 in P3, consistently suggesting that biomass burning is the dominant source of anhydrosugars throughout the sampling period. The L/M radio is the smallest in P2, indicating a relatively significant contribution from biomass burning compared with the other periods. As shown in Figure 6D, the linear correlation between levoglucosan and the ion tracer K^+^ from biomass burning is the strongest in P2 (R^2^ = 0.73) compared with P1 (R^2^ = 0.63) and P3 (R^2^ = 0.26). In the same way, levoglucosan also has significant linear correlations with PM_2.5_ (R^2^ = 0.62), OC (R^2^ = 0.77), and WSOC (R^2^ = 0.73) in P2, the correlations are lower in P1 (R^2^ = 0.44, 0.73, and 0.73 with PM_2.5_, OC, and WSOC), and P3 exhibits the weakest correlations (R^2^ = 0.37, 0.41, and 0.35 with PM_2.5_, OC, and WSOC). The above results prove that biomass burning has the most significant contribution to PM_2.5_ in P2, followed by P1, with P3 showing the least influence. Furthermore, previous studies have confirmed that NOx can be released along with particles from biomass burning, which then forms NO_3_^−^ during the atmospheric aging process.^44^^,^^45^ The correlation between NO_2_, NO_3_^−^, and levoglucosan also reaches maximum values of 0.60 and 0.56 in P2 (Figures 6E–6F), inferring that substantial contributions from biomass burning are also an essential source of increased NO_3_^−^ in P2.

Primary sugars predominantly originate from biogenic sources, including pollen, fruits, and leaves of vegetation.^46^ The concentrations of primary sugars are 13.10 ± 5.64 ng m^−3^, 18.41 ± 10.67 ng m^−3^, and 14.39 ± 7.25 ng m^−3^ in P1, P2, and P3, accounting for 2.76% ± 1.14%, 1.76% ± 0.61%, and 1.87% ± 0.97% of the total detected organic compounds, respectively. Sugar alcohols are mainly associated with airborne fungal spores and decomposing leaf litter.^47^ The concentrations of sugar alcohols are 7.80 ± 6.46 ng m^−3^, 11.83 ± 7.94 ng m^−3^, and 7.92 ± 4.73 ng m^−3^ in P1, P2, and P3, accounting for 1.49% ± 0.74%, 1.08% ± 0.31%, and 0.99% ± 0.37% of the total detected organic compounds, respectively. The sampling period occurs in winter, and the overall concentrations of primary sugars and sugar alcohols are low due to vegetation litter, resulting in a relatively small contribution to PM_2.5_.

#### Lipid compounds

**Figure 7:**
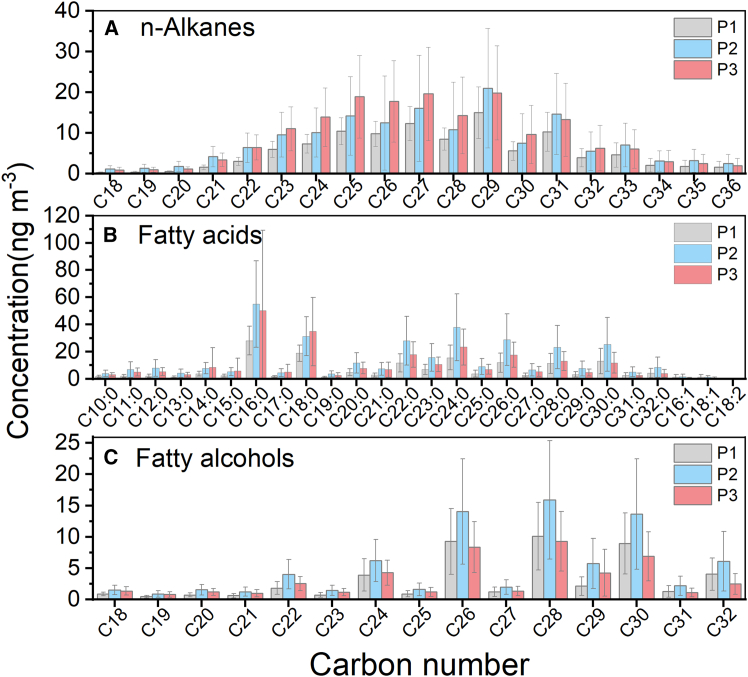
Molecular distribution of lipid compounds Molecular distributions of n-alkanes (A), fatty acids (B), and fatty alcohols (C). Error bars represent SDs of the averages from measurements.

The molecular distributions of lipid compounds, including n-alkanes, fatty acids, and fatty alcohols, are shown in Figure 7. The concentrations of n-alkanes are 105.64 ± 39.38 ng m^−3^, 153.19 ± 107.43 ng m^−3^, and 171.62 ± 94.82 ng m^−3^, in P1, P2, and P3, accounting for 21.32% ± 5.26%, 14.33% ± 4.73%, and 21.27% ± 7.38% of the total detected organic compounds, respectively. The molecular distribution of n-alkanes (Figure 7A) shows that they have considerable concentrations at both high molecular weight (HMW, >C_26_) and low molecular weight (LMW, ≤C_26_), suggesting that they can be derived from both terrestrial plant wax (main source of HMW n-alkanes) and fossil fuel emissions (main source of LMW n-alkanes).^40^^,^^48^ Throughout the entire sampling period, the carbon preference index ([CPI] odd/even) of n-alkanes is close to 1, confirming that their primary derivation is from fossil fuel burning. Previous studies have confirmed that when n-alkanes originate from fossil fuel emissions, the CPI value is near unity, whereas a CPI value close to 10 indicates a plant wax origin.^49^^,^^50^ Nevertheless, there are slight variations across the three periods, with P2 having the highest CPI (1.56 ± 0.17) value and P3 (1.31 ± 0.12) having the lowest, indicating a relatively higher contribution of plant sources in P2, whereas a greater contribution from fossil fuel sources in P3. The calculated percentages of plant wax n-alkanes further support the earlier viewpoint,^51^ with values of 19.39% ± 3.58%, 21.56% ± 5.31%, and 13.62% ± 4.31% in P1, P2, and P3, respectively.

The concentrations of fatty acids are 159.84 ± 77.06 ng m^−3^, 347.92 ± 210.80 ng m^−3^, and 257.05 ± 154.14 ng m^−3^ in P1, P2, and P3, and make up 30.29% ± 4.40%, 32.09% ± 4.37%, and 30.37% ± 6.49% of the total detected organic compounds, respectively. Similar to n-alkanes, HMW fatty acids (≥C_20_) are derived from terrestrial plant waxes, whereas LMW fatty acids (<C_20_) primarily originate from fossil fuel emissions.^48^^,^^50^ The LMW/HMW ratio of fatty acids is the lowest at P2 (0.70 ± 0.24) and highest at P3 (0.92 ± 0.48), proving P2 has a biological source advantage, whereas P3 has a fossil fuel source advantage, resembling n-alkanes. The concentrations of fatty alcohols are 47.19 ± 24.59 ng m^−3^, 78.26 ± 46.75 ng m^−3^, and 47.47 ± 22.50 ng m^−3^ in P1, P2, and P3, accounting for 8.79% ± 1.66%, 7.26% ± 1.85%, and 5.87% ± 1.60% of the total detected organic compounds, respectively. The HMW fatty alcohols (≥C_20_) have an obvious concentration advantage, which primarily originates from higher plant waxes and loess deposits. All lipid compounds exhibited similar change rules in molecular distribution characteristics, showing a higher concentration of HMW lipid compounds in P1 and P2, but a higher concentration of LMW lipid compounds in P3. As shown in Figures 6I–6K, lipid compounds showed a stronger linear correlation with levoglucosan in P1 and P2 than in P3, since HMW lipid compounds mainly come from higher plants, which can also be released during biomass burning. Notably, the concentration of fatty acids dominates the total lipid compounds, with a significant increase, and shows the strongest correlation with levoglucosan (R^2^ = 0.79) in P2, proving more powerfully a relatively considerable contribution from biomass burning to PM_2.5_ during this period.

#### PAHs and hopanes

PAHs predominantly originate from the incomplete combustion of coal, biofuels, and vehicular fuels.^24^ The concentrations of PAHs are 4.27 ± 2.40 ng m^−3^, 9.44 ± 6.08 ng m^−3^, and 7.38 ± 7.59 ng m^−3^ in P1, P2, and P3, making up 0.79% ± 0.26%, 0.90% ± 0.27%, and 0.85% ± 0.51% of the total detected organic compounds, respectively. Source identification of PAHs can be achieved using diagnostic ratios, such as Flu/(Flu+Pyr) (fluoranthene/(fluoranthene+pyrene)) and IP/(IP + BghiP) (indeno[123-cd]pyrene/(indeno[123-cd]pyrene+benzo(ghi)perylene)), as established in previous research. The reported values of Flu/(Flu+Pyr) and IP/(IP + BghiP) are 0.39–0.42 and 0.24–0.35 in the vehicles and 0.51–0.58 and 0.5–0.64 in wood, rice straw, grass, and coal.^52^^,^^53^^,^^54^ During the sampling period, the Flu/(Flu+Pyr) ratios consistently exceed 0.5 and IP/(IP + BghiP) values are also around 0.5, closely matching those of coal and biomass burning sources, which is mainly due to the significant use of coal and wood for heating and daily activities in the surrounding rural areas. Meanwhile, the sampling site is located away from heavily trafficked urban areas, with a relatively minor contribution from motor vehicle emissions. Moreover, the concentration percentage of PAHs in total detected organic compounds in P2 is higher than in P1 and P3, which can be attributed to fewer motor vehicle activities and more coal/biomass consumption for cooking and heating during the Xi’an lockdown.

The concentrations of hopanes are 1.09 ± 0.57 ng m^−3^, 1.72 ± 0.91 ng m^−3^, and 1.74 ± 0.99 ng m^−3^ in P1, P2, and P3, accounting for 0.21% ± 0.08%, 0.17% ± 0.05%, and 0.22% ± 0.07% of the total detected organic compounds, respectively. Hopanes are primarily derived from unburned lubricating oil residues in automobile exhaust emissions. In regions where coal or biomass is the primary energy source for residential use, hopanes may also originate from emissions resulting from coal and biomass burning.^55^^,^^56^ The C_29_αβ/C_30_αβ ratio helps distinguish between coal combustion and traffic emissions. Studies have indicated that the main hopanes emitted from almost all coal combustion are C_29_αβ (C_29_αβ/C_30_αβ>1), whereas the main hopanes in automobile exhaust are C_30_αβ (C_29_αβ/C_30_αβ<1).^57^ Throughout the entire sampling period, the C_29_αβ/C_30_αβ ratio exceeded 1, indicating a predominance of coal combustion. Furthermore, the C_30_αβ/C_30_βα ratio can serve as another indicator to distinguish emission sources. Values below 2.4 generally indicate coal and biomass burning, whereas ratios exceeding 4 suggest vehicular exhaust contributions.^58^ Notably, all C_29_αβ/C_30_αβ ratios are below 2.4 during the entire sampling duration, aligning with coal and biomass burning sources. Moreover, hopanes exhibit a strong linear correlation with PAHs, with R^2^ of 0.62, 0.77, and 0.72 in P1, P2, and P3, respectively. The above evidence proves that hopanes and PAHs originate from similar sources. Especially in P2, the highest R^2^ suggested a more unified emissions source, likely coal and biomass burning during lockdown.

#### Aromatic acids and dicarboxylic acids

**Figure 8:**
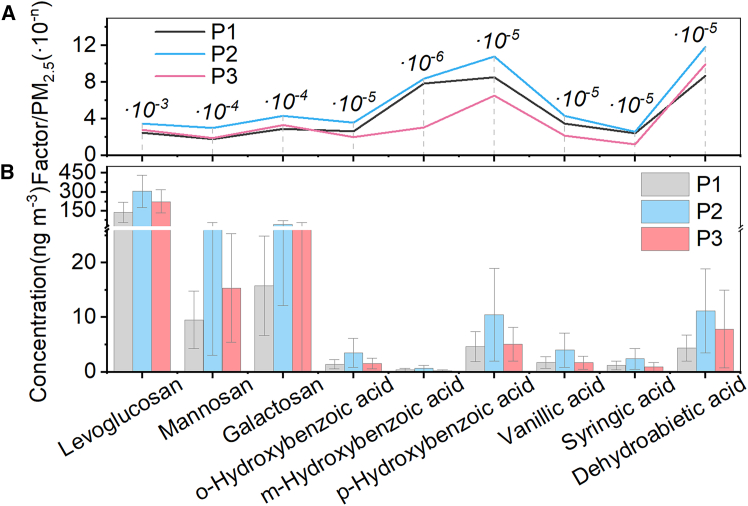
Mass concentrations and PM2.5 contributions of biomass-burning tracers The proportion (A) and the concentration (B) of biomass-burning tracers in PM_2.5_. Error bars in (B) represent SDs of the averages from measurements.

The concentrations of aromatic acids are 13.81 ± 7.23 ng m^−3^, 32.24 ± 21.70 ng m^−3^, and 17.40 ± 11.24 ng m^−3^ in P1, P2, and P3, accounting for 2.61% ± 0.56%, 3.02% ± 0.79%, and 2.17% ± 0.92% of the total detected organic compounds, respectively. Compared with P1 and P3, the concentration percentages in P2 are increased, which may be because these aromatic acids have a similar source as anhydrosugars, mainly derived from lignin combustion in cork, hardwood, and herbaceous plants.^59^^,^^60^ The previous discussion on anhydrosugars has confirmed the enhanced contribution of biomass burning to PM_2.5_ during lockdown. As shown in Figure 8, the concentrations of aromatic acids and anhydrosugars significantly increased in P2, and their proportions in PM_2.5_ are also higher than those in other periods, which once again confirms the previous inference.

The concentrations of dicarboxylic acids are 8.95 ± 3.74 ng m^−3^, 24.48 ± 16.63 ng m^−3^, and 17.57 ± 8.75 ng m^−3^ in P1, P2, and P3, respectively. At the same time, the proportion of them among total detected organic compounds is recorded as 1.77% ± 0.36% in P1, which is significantly lower than the levels in P2 (2.39% ± 1.02%) and P3 (2.35% ± 1.10%). Previous research indicates that dicarboxylic acids predominantly form through secondary processes rather than direct emissions, suggesting significant secondary formation during P2 and P3.^61^ The M/F (maleic acid/fumaric acid) ratio serves as an indicator of the intensity of photochemical oxidation, as maleic acid, derived from aromatic hydrocarbon oxidation, can undergo photochemical isomerization to form fumaric acid.^62^^,^^63^ The conversion efficiency is heavily influenced by atmospheric oxidant levels, ambient temperature, and solar radiation intensity.^64^^,^^65^ The M/F ratios are 0.33 ± 0.16 in P1, 0.27 ± 0.13 in P2, and 0.43 ± 0.53 in P3, much lower than the M/F ratios detected in summer in Guanzhong Plain, which are from 1.1 to 1.4.^66^ A lower M/F value indicates that the photochemical oxidation formation is very weak during the sampling period, likely due to low temperature and weak solar radiation in winter. The previous chapter confirms that the secondary formation of NO_3_^−^ and SO_4_^2−^ primarily occurs through liquid-phase oxidation. As shown in Figures 6M–6P, succinic acid has some correlation with NO_3_^−^ and SO_4_^2−^ across all three periods, as well as a good correlation with ALWC, but is not correlated with O_3_-8h, suggesting that succinic acid formation also primarily arises from liquid-phase oxidation. From the above discussion, it can be inferred that the significant increase in dicarboxylic acids in P2 and P3 is mainly due to enhanced liquid-phase oxidation.

### Cause of heavy PM_2.5_ pollution during Xi’an lockdown

**Figure 9:**
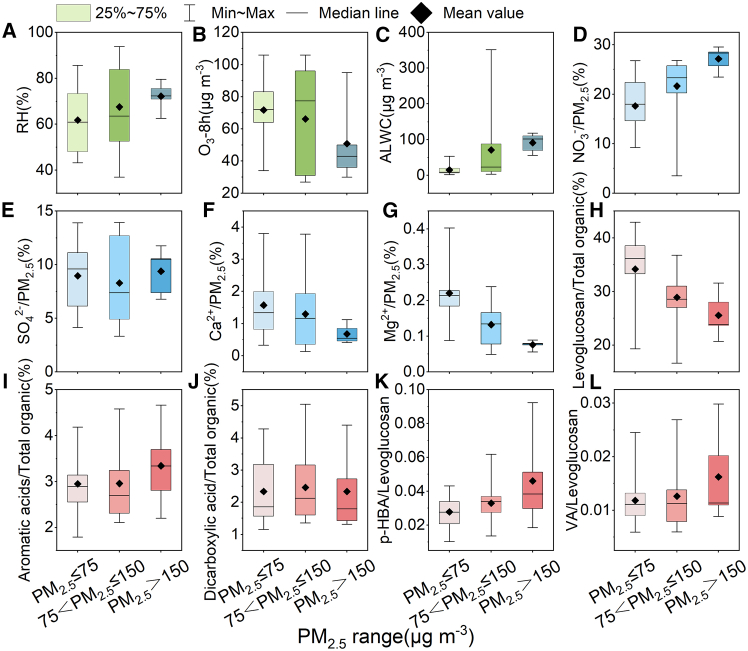
Changes in key indicators across three pollution stages in P2 The RH(%) (A), concentrations of O_3_-8h and ALWC (B, C), proportion of partially WSIIs in PM_2.5_ (D–G), proportion of partially organic compositions in total detected organic compounds (H–J), and the ratios of *p*-HBA/Levo (K) and VA/Levo (L) in “clean,” “light pollution,” and “severe pollution” periods during P2.

During the Xi’an lockdown period (P2), human activities were strictly restricted, leading to a significant reduction in emissions of precursor pollutants. However, the concentrations of PM_2.5_ have risen instead of decreased, even exceeding 200 μg m^−3^ in severe pollution events. In the previous discussion, biomass burning and secondary formation through liquid-phase oxidation have been proved to be essential contributors to PM_2.5_ during this period. To thoroughly analyze the causes of heavy pollution, the lockdown period is divided into three phases according to PM_2.5_ concentrations: the “clean” period (PM_2.5_ ≤ 75 μg m^−3^), the “light pollution” period (75<PM_2.5_ ≤ 150 μg m^−3^), and the “severe pollution” period (PM_2.5_ > 150 μg m^−3^) for further discussion (Figure 9).

During the clean period, the atmospheric conditions include RH = 61.81% ± 15.16%, ALWC = 15.12 ± 14.73 μg m^−3^, and average concentration of PM_2.5_ = 51.17 ± 12.94 μg m^−3^, all of which are similar to P1. Although the NOR (0.20 ± 0.09), SOR (0.19 ± 0.08), and the proportions of SNA in PM_2.5_ (35.32 ± 11.74%) are slightly higher than in P1, they remain significantly lower than the levels recorded during the “light pollution” and “severe pollution” periods (see Table S1). Thus, the atmospheric PM_2.5_ is primarily attributed to direct emissions, similar to P1, with relatively weak liquid-phase oxidation occurring compared with the other periods in P2. Comparing the molecular composition of PM_2.5_ during the “clean” period with that during P1 allows for the observation of changes in direct emission sources caused by the lockdown. Due to restrictions on urban traffic and industrial activity, the concentrations of pollution precursors NO_2_ and SO_2_ decreased by about 35.20% and 13.09% compared with P1. The proportions of Ca^2+^ and Mg^2+^ in PM_2.5_ then decreased to 1.58% ± 1.04% and 0.22% ± 0.07%, respectively. However, the proportions of ion tracer K^+^ in PM_2.5_ (0.73% ± 0.14%) and organic tracer levoglucosan in total detected organic compounds (34.16% ± 6.40%) of biomass burning emissions increased significantly, confirming the inference that biomass burning emissions in suburban regions increased significantly during the lockdown.

As pollution intensifies, the proportions of Ca^2+^ and Mg^2+^ in PM_2.5_ decrease significantly, dropping from 1.30% ± 1.01% and 0.13% ± 0.05% during the “light pollution” periods to 0.68% ± 0.30% and 0.08% ± 0.01%, respectively, under the “severe pollution” conditions. In contrast, the proportion of K^+^ increases slightly, rising from 0.91% ± 0.23% (“light pollution”) to 0.95% ± 0.12% (“severe pollution”). Meanwhile, the proportions of SNA in PM_2.5_ show a marked increase with worsening pollution, ranging from 35.32% ± 11.74% to 40.06% ± 12.86% and then to 49.17% ± 6.38%. Correspondingly, NOR and SOR reach 0.48 ± 0.05 and 0.45 ± 0.09, respectively, during the “severe pollution” period. Among the SNA components, the contribution of NO_3_^−^ to PM_2.5_ increases progressively with pollution intensity, whereas the contribution of SO_4_^2−^ remains relatively stable. The proportion of NO_3_^−^ in PM_2.5_ rises from 17.65% ± 5.56% to 21.65% ± 6.28% and then to 27.14% ± 2.48%, whereas the proportion of SO_4_^2−^ in PM_2.5_ fluctuates between 8.96% ± 3.35%, 8.29 ± 3.79%, and 9.39 ± 2.18%, corresponding to transitions from “clean” to “light pollution” to “severe pollution” periods. The varying trends in the proportions of WSIIs in PM_2.5_ suggest that the formation of heavy pollution weather mainly results from the contribution of biomass burning and secondary aerosol formation. In particular, the promoting effect of NO_3_^−^ on heavy pollution occurrence is significantly more potent than that of SO_4_^2−^ in secondary formation.

Further discussion revealed that the proportion of levoglucosan in total detected organic compounds gradually decreases from “clean” to “light pollution” to “severe pollution” period, ranging from 34.16% ± 6.40% to 28.89% ± 4.75% and then to 25.55% ± 4.26%. Interestingly, aromatic acids, which share similar sources with levoglucosan, exhibited an opposite trend, the proportions of them in total detected organic compounds increasing from 2.95% ± 0.73% (“clean”) to 2.96% ± 0.83% (“light pollution”) and further to 3.34% ± 0.93% (“severe pollution”), which may be attributed to the fact that anhydrosugars come from direct emissions of biomass burning, whereas the aromatic acids are primarily produced through the aging of biomass burning aerosols.^60^ The ratios of VA/Levo (vanillic acid/levoglucosan) and *p*-HBA/Levo (*p*-hydroxybenzoic acid/levoglucosan) can be used as indicators for the aging of aerosols from biomass burning in the field study.^67^ The ratios are 0.012 ± 0.005 and 0.028 ± 0.009 during the “clean” period, 0.013 ± 0.006 and 0.033 ± 0.012 during the “light pollution” period, 0.016 ± 0.009 and 0.046 ± 0.028 during the “severe pollution” period, confirming the promoting effect of biomass-burning SOA on the formation of heavy pollution weather.

For other detected SOA components, such as dicarboxylic acids, the proportions of them in total detected organic compounds are 2.34 ± 0.99% in the “clean” period, increasing to 2.46% ± 1.03% in the “light pollution” period but decreasing to 2.33% ± 1.28% in the “severe pollution” period. A reasonable speculation is that during the accumulation of pollution, the ability of atmospheric aging continues to strengthen and long-chain dicarboxylic acids are gradually oxidized to short-chain dicarboxylic acids. During the “light pollution” period, dicarboxylic acids may first be oxidized into slightly-higher-molecular-weight products, and then further oxidized into lower-molecular-weight products during the “severe pollution” period. However, because the SOA components detected do not contain short-chain dicarboxylic acids such as oxalic acid, the percentage of total dicarboxylic acid concentration shows a trend of first increasing and then decreasing. Succinic acid is a major precursor of oxalic acid during the atmospheric aging process.^68^ The proportion of succinic acid in total detected organic compounds also shows a trend of first increasing and then decreasing (0.84% ± 0.51%, 0.95% ± 0.59%, and 0.78% ± 0.58% in the three pollution phases), confirming our speculation about the aging of the atmosphere. The M/F values are 0.28 ± 0.08, 0.29 ± 0.19, and 0.20 ± 0.04 in the “clean,” “light pollution,” and “severe pollution” periods, respectively. Such a low value indicates that the effect of photochemical oxidation on the concentration of dicarboxylic acids is minimal. Thus, the atmospheric aging process of dicarboxylic acids should mainly occur in the liquid phase.

### PM_2.5_ source apportionment by PMF

**Figure 10:**
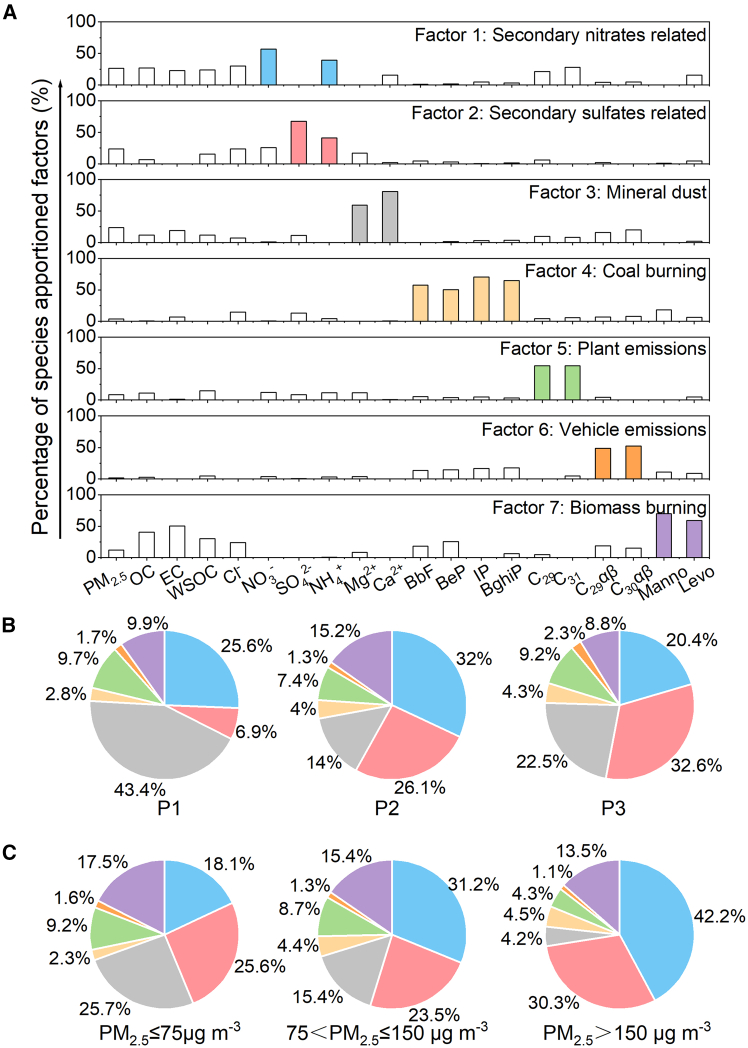
Source apportionment of PM2.5 by the PMF model Proportions of the chemical species appointed to the seven factors resolved by PMF (A); source apportionment for PM_2.5_ in P1, P2, and P3 (B); and source apportionment for PM_2.5_ in stages PM_2.5_ ≤ 75 μg m^−3^, 75<PM_2.5_ ≤ 150 μg m^−3^, and PM_2.5_ > 150 μg m^−3^ of P2 (C).

The sources of PM_2.5_ were determined using the Positive Matrix Factorization (PMF) 5.0 model, developed by the United States Environmental Protection Agency (EPA). Two-factor to nine-factor PMF models have been simulated, and a seven-factor model was selected because the Q value did not show a significant decrease starting from the seven-factor model.^69^ The values of Q_true_ and Q_robust_ were convergent in the seven-factor model (Q_robust_ = 648.2, Q_true_ = 648.9), and the average correlation coefficient between input and model values is 0.98. These results confirmed that the seven-factor model fits the input data. As illustrated in Figure 10A, seven factors were identified. **Factor 1** was identified as a secondary nitrate-related source, based on the high contributions of NO_3_^−^ and NH_4_^+^. **Factor 2,** characterized by high loadings of SO_4_^2−^ and NH_4_^+^, was attributed to a secondary sulfate-related source. **Factor 3** was dominated by Ca^2+^ and Mg^2+^, ions typical of flowing dust, and was therefore assigned to a mineral dust source. **Factor 4** was dominated by the four selected PAHs, which are primarily emitted from fossil fuel combustion, identifying it as a coal-burning source. **Factor 5** demonstrated high loadings for C_29_ and C_31_ n-alkanes, indicating a plant emissions source. **Factor 6** was dominated by C_29_αβ and C_30_αβ hopanes, which are established tracers for vehicle exhaust, and was thus classified as vehicle emissions. **Factor 7** was characterized by elevated levels of levoglucosan and mannosan, typical indicators of biomass burning. High OC, EC, WSOC, and Cl^−^ loadings further confirmed this factor as biomass burning emissions.

The contribution of each source to PM_2.5_ concentration during three typical periods is depicted in Figure 10B. Secondary nitrates-related, secondary sulfates-related, mineral dust, and biomass burning are relatively essential sources of emissions. During P1, mineral dust sources (43.4%) are the most important source of PM_2.5_, possibly due to the busy construction activities and road traffic in this period, as well as the semi-arid climate and unique topography in Guanzhong Plain. Secondary formation sources include secondary nitrates-related sources (25.6%) and secondary sulfates-related sources (6.9%), which also make substantial contributions to PM_2.5_ but are much lower than the contributions in P2 and P3. Finally, the contribution of biomass-burning sources is 9.9% in P1. During P2, the secondary nitrates-related sources (32%) become the most crucial source, and the contribution of the secondary sulfates-related sources (26.1%) also significantly increased, mainly due to the significant enhancement of liquid-phase oxidation generation with the increase of RH (65.93 ± 15.47%). The contribution of biomass-burning sources rose to 15.2%, driven by increased biomass consumption for cooking and heating following the enforcement of strict regulations on outdoor activities. In contrast, the mineral dust sources (14%) significantly decreased due to reduced construction activities and road traffic. During P3, secondary nitrates-related sources (20.4%) and secondary sulfates-related sources (32.6%) remain the important sources of PM_2.5_. Secondary sulfates-related sources have increased significantly since metal ions from fireworks emissions can catalyze the secondary formation of SO_4_^2−^. As residents restore normal production and living order, mineral dust sources (22.5%) significantly increase and biomass burning sources (8.8%) decrease relatively. In all three periods, coal burning, plant emissions, and vehicle emissions are comparatively minor contributors to PM_2.5_. Among them, vehicle emissions are significantly lower than coal burning and plant emissions remain relatively stable.

Figure 10C shows the results of a detailed source apportionment of PM_2.5_ in “clean,” “light pollution,” and “severe pollution” periods during lockdown. Comparing the source contribution of the “clean” period and the “normal” period (P1), the contributions of primary sources, such as biomass burning (17.5%), increased by 76.77% and mineral dust (25.7%) decreased by 40.78% compared with P1. The contributions of mineral dust sources continue to decline from the “clean” period to the “light pollution” period and then to the “severe pollution” period, being 25.7%, 15.4%, and 4.2%, respectively. Similarly, the contributions of biomass burning sources decreased from 17.5% (“clean” period) to 15.4% (“light pollution”), ultimately decreasing to 13.5% during the “severe pollution” period. On the contrary, the contributions of secondary sources show an upward trend with the intensification of PM_2.5_ pollution. Among them, the contributions of secondary nitrates-related sources increased more significantly due to the joint contribution of biomass burning and liquid-phase oxidation.

## Summary and conclusions

Daily observations of atmospheric PM_2.5_ were conducted at a background area near a megacity in the Guanzhong Plain, China, from November 24, 2021, to February 24, 2022. The measurement data included carbonaceous fractions, WSIIs, and organic compounds in PM_2.5_ during three typical emission scenarios (“normal,” “lockdown,” and “festival”). The results showed that the molecular composition and sources of PM_2.5_ in the background site differ significantly from those in urban areas. Compared with urban areas, the contributions of motor vehicle and coal-burning emissions to PM_2.5_ at background sites are relatively small, whereas biomass burning and secondary formation sources contribute more significantly to PM_2.5_ levels. During the “normal” period, direct emissions contribute significantly to PM_2.5_ concentration under low RH conditions, with a high proportion of mineral dust markers Ca^2+^ and Mg^2+^ in PM_2.5_. Source apportionment results by PMF also showed that mineral dust sources contributed over 40% of PM_2.5_. During the “lockdown” period, although the concentration of NO_2_ and SO_2_ obviously decreased compared with the “normal” period, PM_2.5_ increased from 59.36 ± 33.03 μg m^−3^ to 97.61 ± 53.70 μg m^−3^. The concentration of SNAs (SO_4_^2−^, NH_4_^+^, and NO_3_^−^) increased the most among the measured chemical compositions and showed a significant correlation with RH and ALWC, suggesting the crucial role of liquid-phase oxidation. The proportion of biomass burning markers, i.e., Cl^−^, K^+^, anhydrosugars, and aromatic acids in PM_2.5_, notably rose, demonstrating the significant contribution of biomass burning. The molecular distribution of WSIIs and organic compounds further confirmed the enhanced secondary formation through liquid-phase oxidation in severe pollution events (PM_2.5_ > 150 μg m^−3^). Source apportionment results by PMF indicated that the contribution of biomass-burning sources to PM_2.5_ increased by over 70% compared with the “normal” period. Secondary formation sources account for more than 50% of PM_2.5_ levels in the “lockdown” period and even reach 72% in the “severe pollution” events. During the “festival” period, fireworks-related species, i.e., Mg^2+^, Cl^−^, and K^+^, showed higher proportions in PM_2.5_ compared with other periods. SOR increased more rapidly than NOR, indicating that metal ions from fireworks emissions could accelerate the liquid-phase formation of sulfate. PMF analysis results display secondary sulfates-related sources contributing to 33% of PM_2.5_. The results highlight the significant role of the synergistic effects between biomass/fireworks burning and liquid-phase oxidation in the formation of heavy pollution events and point out that unbalanced emission reductions may worsen the interactions between pollutants due to atmospheric aging and cross-regional transport. Thus, our findings underscore that effectively addressing air pollution requires a coordinated and balanced strategy for controlling multiple pollutants. Although the current state of air pollution varies across different regions, future emission control strategies must not only prioritize reductions in emissions from urban industries and transportation but also impose practical constraints on pollutant emissions from households and personal activities, particularly in rural areas.

### Limitations of the study

This study has several limitations. First, the concentrations of NH_3_, HNO_3_, and HCl in the gas phase were unavailable for our analysis, leading to some uncertainty when using the ISORROPIA-II model to estimate pH. Second, the lack of measurements for metals such as Fe and Cu in PM_2.5_ samples restricts a comprehensive assessment of the metal-catalyzed formation of sulfate in the liquid phase. Third, our research was based on daily PM_2.5_ samples (10:00 a.m. to 09:00 a.m. the following day), which may obscure short-term variations in aerosol composition, limiting the accurate characterization of real-time atmospheric aging processes. Finally, the field observations for this study were conducted at a single urban background site. Although the findings can provide certain scientific guidance for refining future air pollution control strategies, a more comprehensive and accurate formulation of regional pollution control measures will require additional field observations at representative locations.

## Resource availability

### Lead contact

Further information and requests for resources should be directed to and will be fulfilled by the lead contact, Jianjun Li (lijj@ieecas.cn).

### Materials availability

This study did not generate new unique reagents.

### Data and code availability

• **Data:** All raw data have been deposited at Mendeley at (https://data.mendeley.com/datasets/fnwjyg6fwh/1) and are publicly available as of the date of publication.
• **Code:** This paper does not report the original code.
• **Additional information:** Any additional information required to reanalyze the data reported in this paper is available from the lead contact upon request.

## Acknowledgments

This work was jointly supported by the program from 10.13039/501100014888State Key Laboratory of Loess Science, Institute of Earth Environment, CAS (SKLLOG2307), the Natural Science Basic Research Program of Shaanxi (2025JC-YBQN-450), and 10.13039/501100001809National Natural Science Foundation of China (No. 42407156). J.L. also acknowledges the support of the Youth Innovation Promotion Association CAS.

## Author contributions

Conceptualization, data curation, formal analysis, funding acquisition, software, validation, visualization, writing-original draft, and writing-review and editing, Y.L.; data curation, formal analysis, validation, and writing-review and editing, X. G.; investigation, validation, and visualization, Y.Z.; data curation, software, writing-review and editing, Y.C.; investigation and visualization, Y.J.; formal analysis and validation, W.Q.; methodology, funding acquisition, and writing-review and editing, M.S.; formal analysis, L.L.; investigation, Q. W.; supervision and writing-review and editing, W.D.; conceptualization, funding acquisition, methodology, project administration, resources, supervision, and writing-review and editing, J.L.

## Declaration of interests

The authors declare no competing interests.

## STAR★Methods

### Key resources table

**Table:** 

REAGENT or RESOURCE	SOURCE	IDENTIFIER
**Deposited data**		

PM_2.5_ components	This paper	https://data.mendeley.com/datasets/fnwjyg6fwh/1
Gaseous pollutants	Air automatic monitoring station in Zhouzhi County, Shaanxi, China	N/A
Meteorological parameters	National Observation and Research Station of Regional Ecological Environment Change and Comprehensive Management in the Guanzhong Plain, Shaanxi, China	N/A
Digital elevation model of Shaanxi, China	National Aeronautics and Space Administration	https://search.earthdata.nasa.gov/search

**Chemicals, peptides, and recombinant proteins**		

Chemical analytical standards	Sigma-Aldrich	https://www.sigmaaldrich.cn
Organic solvents (methanol, dichloromethane, n-hexane)	Fisher	https://www.thermofisher.cn
N,O-bis(trimethylsilyl) trifluoroacetamide	Sigma-Aldrich	https://www.sigmaaldrich.cn

**Software and algorithms**		

PMF model (PMF 5.0)	United States Environmental Protection Agency	https://cfpub.epa.gov/si/si_public_record_report.cfm?Lab=NERL&dirEntryId=308292
ISORROPIA-II model	Fountoukis & Nenes, 2007	https://doi.org/10.5194/acp-7-4639-2007
HYSPLIT model	National Oceanic and Atmospheric Administration	http://ready.arl.noaa.gov/HYSPLIT_traj.php
Origin 2022	OriginLab Corporation	https://www.originlab.com
ArcGIS 10.8	Environmental Systems Research Institute, Inc.	https://www.esri.com

**Other**		

Quartz fiber filter	Whatman	http://whatman.utopbio.com
Mid-volume air sampler (HC-1010)	Qingdao Hecheng Environmental Protection Technology Co., China	https://www.qdhchb.cn
Carbon analyzer (MODEL 2001)	Atmoslytic	http://www.bmet.cn
Total organic carbon analyzer (TOC-L CPH)	Shimadzu	https://www.shimadzu.com.cn
Ion chromatography (Metrohm 940)	Metrohm	https://www.metrohm.cn
GC/EI-MS (Agilent 7890A/5975C)	Agilent	https://www.agilent.com.cn

### Experimental model and study participant details

This study did not involve experimental models or study participants.

### Method details

#### Sample collection

PM_2.5_ sampling was carried out at Tianyu observation station (34°3′39″N, 108°20′37″E; 503 m a.s.l.), part of the National Observation and Research Station of Regional Ecological Environment Change and Comprehensive Management in the Guanzhong Plain, Shaanxi, China (Figure S1). The site is situated along the northern foothills of the Qinling Mountains, approximately 60 km southwest of Xi’an, with no significant nearby sources of anthropogenic air pollution. From November 24, 2021, to February 24, 2022, daily PM_2.5_ samples (10:00 to 09:00 the following day) were collected using a mid-volume air sampler (HC-1010, Qingdao Hecheng Environmental Protection Technology Co., China) with pre-baked (450 °C, 5 h) quartz fiber filters (Whatman, QMA, USA) at an airflow rate of 100 L min^−1^. A total of 89 samples were obtained, individually wrapped in aluminum foil bags, and stored at −20 °C until analysis. For quality control, field blank samples were collected before and after sampling by loading a pre-baked filter into the sampler for 10 min without drawing any air. All reported data were corrected using the average concentration derived from the two field blank samples.

#### Carbonaceous components and WSIIs analysis

A 0.526 cm^2^ filter sample was analyzed for OC and EC content using a DRI Model 2001 carbon analyzer following the IMPROVE-A thermal-optical reflectance protocol. The analyzer was calibrated with a known quantity of CH_4_ every day. One sample was randomly selected from 10 samples and reanalyzed. Differences determined from the replicate analyses were <5% for TC and <10% for OC and EC. All carbonaceous components were corrected by the field blanks (less than 10% of those in the ambient samples). MDLs of TC, OC, and EC are 0.44, 0.41, and 0.03 μg cm^−2^, respectively. The mass concentrations of all the detected carbonaceous components were significantly higher than the MDLs. The aqueous extract of filter samples was used for the detection of WSOC and WSIIs, which was obtained by ultrasonication (15 min each, repeated three times) of one-quarter of the filter samples in 30 mL Milli-Q water, followed by PTFE filtration to remove particles and debris. WSOC was determined via a total organic carbon (TOC) analyzer (TOC-L CPH, Shimadzu, Japan). WSIIs, including NO_3_^−^, SO_4_^2−^, Cl^−^, NH_4_^+^, Na^+^, K^+^, Mg^2+^, and Ca^2+^, were analyzed by ion chromatography (Metrohm 940 Professional IC Vario). A fixed amount of lithium bromide was added to the eluent to increase the accuracy of the WSIIs measurement. One sample was randomly selected from 10 samples and reanalyzed, and the collection efficiencies of the target species were higher than 89%. All WSIIs were corrected by the field blanks (less than 10% of those in the ambient samples). MDLs of NO_3_^−^, SO_4_^2−^, Cl^−^, NH_4_^+^, Na^+^, K^+^, Mg^2+^, and Ca^2+^ are 0.026, 0.027, 0.0087, 0.0010, 0.0005, 0.0011, 0.0008, and 0.0012 μg mL^−1^, respectively. The mass concentrations of all the detected WSIIs were significantly higher than the MDLs. Further methodological details of carbonaceous components and WSIIs analysis can be found in earlier studies.^22^

#### Organic compounds analysis

Organic compounds were analyzed by gas chromatography/electron ionization mass spectrometry (GC/EI-MS, Agilent 7890A GC/5975C MS) following established methods.^70^ Filter samples were ultrasonically extracted with dichloromethane: methanol (2:1, v/v), concentrated by rotary evaporation and nitrogen purge, then derivatized with N,O-bis(trimethylsilyl) trifluoroacetamide (BSTFA) (70 °C, 3 h). GC separation was performed using a DB-5MS capillary column with temperature programming: 50 °C (initial), ramped at 15 °C/min to 120 °C, then 5 °C/min to 300 °C (held for 16 min). The injector temperature was 280 °C (splitless mode), with mass spectra acquired in electron impact (EI) mode (70 eV) from m/z 50–650. GC/EI-MS response factors were determined using authentic standards, with recoveries of 80–120% for all target compounds. All field blanks were subjected to the same extraction and analysis procedures as actual samples, and quantified target compounds in blanks were lower than 5% of ambient sample concentrations. Reported data were adjusted for field blanks but were not corrected for recovery rates. MDLs were calculated based on a signal-to-noise ratio (SNR) approach. Signal-to-noise ratios of 3:1 were considered for the estimations of MDLs in the current study. MDLs were in the range of 0.004–0.122 μg L^−1^ for all organic compounds studied. 95% of the reported concentrations of organic compounds exceeded the MDLs, while data falling below the detection limit are presented using the actual measured values.

#### PMF model analysis

PM_2.5_ sources were determined by the positive matrix factorization (PMF) model.^69^ Details of the methodology can be found on the United States Environmental Protection Agency (EPA) Website (https://cfpub.epa.gov/si/si_public_record_report.cfm?Lab=NERL&dirEntryId=308292). 89 samples and 20 species were used as the input matrix in PMF. The list of input species includes PM_2.5_, OC, EC, WSOC, WSIIs (NO_3_^−^, SO_4_^2−^, NH_4_^+^, Mg^2+^ and Ca^2+^), and selected organic markers (benzo(*b*)fluoranthene (BbF), Benzo(e)pyrene (BeP), indeno(1,2,3-*c*,*d*)pyrene (IP), benzo(ghi)perylene (BghiP), nonacosane (C_29_), hentriacotane (C_31_), 17α(H), 21β(H)-30-norhopane (C_29_αβ), 17α(H), 21β(H)-hopane (C_30_αβ), mannosan, levoglucosan. It is important to note that both K^+^ and anhydrosugars (levoglucosan, mannosan, and galactosan) can be produced through the combustion and pyrolysis of cellulose and hemicellulose. However, K^+^ is not an ideal tracer for biomass burning compared with anhydrosugars since it can also originate from several sources unrelated to biomass burning, including soil dust, sea salt, coal combustion, and fireworks burning. Using K^+^ as an input species in PMF may introduce additional uncertainties into source analysis. Therefore, K^+^ was not included in the PMF analysis of biomass burning factor.

#### ISORROPIA-II model analysis

Aerosol liquid water content (ALWC) was estimated using ISORROPIA-II model, with “forward” mode.^71^ pH was derived from aqueous-phase hydrogen ion activity, which was calculated using Equation 1:

(Equation 1) $$\text{pH}=-\mathrm{lg}\frac{1000\gamma_{H^{+}}H_{\text{air}}^{+}}{\text{ALWC}}$$

$\gamma_{H^{+}}$ is the activity coefficient of H^+^ ($\gamma_{H^{+}}$ = 1), $H_{air}^{+}$ (μg m^−3^) denotes the hydronium ion concentration per volume of air. ALWC (μg m^−3^) is aerosol liquid water content. Due to the unavailability of gas-phase NH_3_, HNO_3_, and HCl concentrations, the model considered only the NO_3_^−^- SO_4_^2-^- Cl^−^- NH_4_^+^- Na^+^- K^+^- Mg^2+^- Ca^2+^ system, potentially introducing a pH underestimation of one unit.^72^^,^^73^

#### HYSPLIT model analysis

The 48-h air mass backward trajectories were calculated by the NOAA Hybrid Single Particle Lagrangian Integrated Trajectory (HYSPLIT) model (http://ready.arl.noaa.gov/HYSPLIT_traj.php) at an arrival height of 100 m above ground level.

### Quantification and statistical analysis

All quantitative and statistical analyses for this study were conducted using Microsoft Excel (Tables S1, S2, and S3). Graphs were created using Origin 2022 (Figures 1, 3, 4, 5, 6, 7, 8, 9, and 10), HYSPLIT model (Figure 2), and ArcGIS 10.8 (Figure S1).

## Supplemental information

Document S1. Figure S1 and Tables S1 and S3

Table S2. Average concentrations of the organic compound classes in PM2.5 samples
