# Supplementary material for: Chemical characteristics of wintertime PM2.5 in background region of northwest China during urban emission reduction
Source: iScience. 2025 Sep 9;28(10):113528. doi: 10.1016/j.isci.2025.113528 (PMC12506562; doi:10.1016/j.isci.2025.113528)
Supplement: Document S1. Figure S1 and Tables S1 and S3 [file mmc1.pdf]

iScience, Volume *28*

Supplemental information

Chemical characteristics of wintertime $PM_{2.5}$ in background region of northwest China during urban emission reduction

Yali Liu, Xiao Guo, Yifan Zhang, Yue Cao, Yingkun Jiang, Weining Qi, Minxia Shen, Lu Li, Qian Wang, Wenting Dai, and Jianjun Li

Supplemental Figure

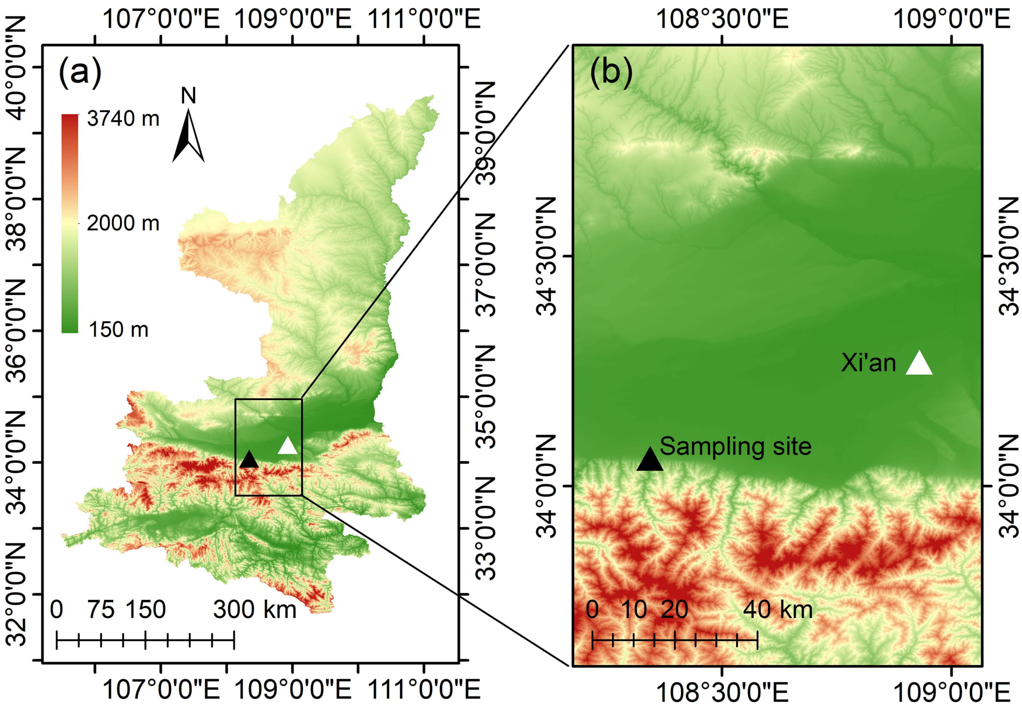

Figure S1. The sampling site.

Supplemental Tables

Table S1. Average concentrations, concentration percentages, and diagnostic ratios of the major chemical compositions in three periods. Data are presented as mean ± standard deviation.

Species	Period 1 (N=26)	Period 2 (N=32)				Period 3 (N=31)
		Average (N=32)	$PM_{2.5}$≤75 μg m^{-3} (N=13)	75＜$PM_{2.5}$≤150 μg m^{-3} (N=14)	$PM_{2.5}$＞150 μg m^{-3} (N=5)	
**Meteorological parameters**						
T (℃)	5.45 ± 1.74	1.63 ± 2.25	1.43 ± 2.87	1.96 ± 1.99	1.22 ± 0.97	1.97 ± 2.01
RH (%)	53.98 ± 13.31	65.93 ± 15.47	61.81 ± 15.16	67.51 ± 17.64	72.22 ± 6.32	69.18 ± 15.09
Wind speed (m s^{-1})	0.74 ± 0.55	0.49 ± 0.36	0.70 ± 0.39	0.39 ± 0.28	0.24 ± 0.16	0.58 ± 0.38
**Chemical compositions**						
SO_2 (μg m^{-3})	15.58 ± 2.94	14.13 ± 2.83	13.54 ± 2.33	14.36 ± 3.27	15.00 ± 2.92	8.48 ± 2.23
CO (mg m^{-3})	1.08 ± 0.27	1.81 ± 0.46	1.46 ± 0.37	1.91 ± 0.30	2.44 ± 0.09	1.24 ± 0.35
O_3-8h (μg m^{-3})	59.73 ± 17.65	66.06 ± 26.29	71.77 ± 19.61	66.21 ± 31.09	50.80 ± 25.82	76.87 ± 23.62
NO_2 (μg m^{-3})	44.04 ± 11.79	34.06 ± 9.72	28.54 ± 7.85	35.64 ± 8.80	44.00 ± 7.97	32.13 ± 11.73
$PM_{2.5}$ (μg m^{-3})	59.36 ± 33.03	97.61 ± 53.70	51.17 ± 12.94	104.01 ± 16.97	200.43 ± 23.33	83.26 ± 28.63
OC (μg m^{-3})	9.04 ± 4.84	15.77 ± 8.68	8.54 ± 2.81	17.39 ± 4.54	30.05 ± 7.69	10.49 ± 3.72
EC (μg m^{-3})	1.59 ± 0.89	2.30 ± 1.30	1.30 ± 0.60	2.51 ± 0.75	4.31 ± 1.29	1.55 ± 0.66
WSOC (μg m^{-3})	5.63 ± 2.70	10.14 ± 5.35	5.62 ± 1.54	11.07 ± 2.87	19.31 ± 3.60	8.14 ± 2.45
OC/EC	5.94 ± 1.15	6.99 ±1.07	6.91 ± 1.13	7.02 ± 1.21	7.08 ± 0.54	7.25 ± 1.71
WSOC/OC	0.65 ± 0.09	0.65 ± 0.07	0.67 ± 0.09	0.64 ± 0.05	0.65 ± 0.07	0.79 ± 0.08
NO_3^- (μg m^{-3})	10.25 ± 11.25	22.29 ± 16.71	9.11 ± 3.77	23.14 ± 8.52	54.21 ± 6.60	17.18 ± 9.84
SO_4^{2-} (μg m^{-3})	2.93 ± 2.03	8.68 ± 6.09	4.56 ± 1.98	8.96 ± 5.03	18.61 ± 3.81	9.74 ± 4.91
NH_4^+ (μg m^{-3})	3.82 ± 4.06	10.54 ± 7.99	4.49 ± 2.12	10.93 ± 4.97	25.19 ± 3.63	8.80 ± 5.01
Cl^- (μg m^{-3})	0.62 ± 0.53	1.67 ± 1.25	0.58 ± 0.23	1.85 ± 0.58	3.99 ± 0.26	1.31 ± 0.67
Na^+ (μg m^{-3})	0.66 ± 0.18	0.69 ± 0.14	0.60 ± 0.09	0.71 ± 0.09	0.91 ± 0.08	1.20 ± 0.21
K^+ (μg m^{-3})	0.42 ± 0.32	0.86 ± 0.56	0.37 ± 0.11	0.95 ± 0.29	1.88 ± 0.19	0.83 ± 0.69
Mg^{2+} (μg m^{-3})	0.14 ± 0.04	0.12 ± 0.04	0.11 ± 0.03	0.13 ± 0.04	0.15 ± 0.03	0.17 ± 0.09
Ca^{2+} (μg m^{-3})	1.49 ± 0.74	1.08 ± 0.75	0.81 ± 0.67	1.24 ± 0.86	1.33 ± 0.49	1.22 ± 0.83
SNA (μg m^{-3})	16.99 ± 17.27	41.52 ± 30.38	18.16 ± 7.65	43.03 ± 17.81	98.02 ± 13.32	35.73 ± 18.82
Total WSII (μg m^{-3})	20.34 ± 18.37	45.95 ± 32.16	20.62 ± 7.55	47.92 ± 17.75	106.28 ± 13.19	40.46 ± 18.95
NOR	0.12 ± 0.09	0.30 ± 0.14	0.20 ± 0.09	0.32 ± 0.12	0.48 ± 0.05	0.27 ± 0.09
SOR	0.11 ± 0.06	0.27 ± 0.15	0.19 ± 0.08	0.29 ± 0.16	0.45 ± 0.09	0.42 ± 0.16
pH	5.48 ± 1.81	4.33 ± 1.10	4.34 ± 1.46	4.38 ± 0.96	4.18 ± 0.09	4.23 ± 0.94
ALWC (μg m^{-3})	11.09 ± 14.63	51.59 ± 72.12	15.12 ± 14.73	71.27 ± 98.09	91.35 ± 27.09	61.28 ± 88.34
**Chemical compositions/$PM_{2.5}$ (%)**						
OC	15.55 ± 2.95	16.46 ± 3.65	16.76 ± 3.53	16.73 ± 4.02	14.89 ± 3.15	12.63 ± 2.01
EC	2.71 ± 0.72	2.43 ± 0.75	2.51 ± 0.72	2.47 ± 0.85	2.13 ± 0.55	1.85 ± 0.55
WSOC	10.03 ± 2.11	10.67 ± 2.11	11.16 ± 2.19	10.60 ± 2.27	9.59 ± 1.09	9.98 ± 1.50
NO_3^-	13.97 ± 8.34	20.89 ± 6.35	17.65 ± 5.56	21.65 ± 6.28	27.14 ± 2.48	19.72 ± 6.47

**SO_4^{2-}**	4.87 ± 1.39	8.73 ± 3.34	8.96 ± 3.35	8.29 ± 3.79	9.39 ± 2.18	11.75 ± 4.78
**NH_4^+**	5.36 ± 3.13	9.94 ± 3.48	8.70 ± 3.31	10.12 ± 3.67	12.64 ± 1.75	10.22 ± 3.83
**Cl^-**	0.95 ± 0.35	1.54 ± 0.51	1.11 ± 0.27	1.76 ± 0.48	2.00 ± 0.15	1.54 ± 0.51
**Na^+**	1.35 ± 0.55	0.88 ± 0.38	1.23 ± 0.32	0.70 ± 0.18	0.46 ± 0.09	1.61 ± 0.61
**K^+**	0.66 ± 0.20	0.84 ± 0.20	0.73 ± 0.14	0.91 ± 0.23	0.95 ± 0.12	0.96 ± 0.67
**Mg^{2+}**	0.28 ± 0.10	0.16 ± 0.08	0.22 ± 0.07	0.13 ± 0.05	0.08 ± 0.01	0.22 ± 0.12
**Ca^{2+}**	2.84 ± 1.10	1.31 ± 0.97	1.58 ± 1.04	1.30 ± 1.01	0.68 ± 0.30	1.57 ± 1.07
**SNA**	24.21 ± 12.31	39.56 ± 12.27	35.32 ± 11.74	40.06 ± 12.86	49.17 ± 6.38	41.70 ± 13.18
**Total WSII**	30.28 ± 11.31	44.29 ± 11.54	40.18 ± 10.71	44.86 ± 12.24	53.34 ± 6.54	47.60 ± 11.54

SNA: SNA=$[NO_3^-]+[SO_4^{2-}]+[NH_4^+]$, the concentration in the formula is the mass concentration of the corresponding substance.

NOR: NOR=$[NO_3^-]/([NO_3^-]+[NO_2])$, the concentration in the formula is the molar concentration of the corresponding substance.

SOR: $[SO_4^{2-}]/([SO_4^{2-}]+[SO_2])$, the concentration in the formula is the molar concentration of the corresponding substance.

Table S3. Average concentrations, concentration percentages, and diagnostic ratios of detected organic compounds in three periods. Data are presented as mean ± standard deviation.

Species	Period 1 （N=26）	Period 2 (N=32)				Period 3 （N=31）
		Average (N=32)	$PM_{2.5}$≤75 μg m^{-3} (N=13)	75＜$PM_{2.5}$≤150 μg m^{-3} (N=14)	$PM_{2.5}$＞150 μg m^{-3} (N=5)	
**Organic compounds**						
**Sugar compounds (ng m^{-3})**	186.29 ± 99.09	407.59 ± 186.47	267.39 ± 110.95	431.84 ± 125.07	704.20 ± 76.16	290.78 ± 116.38
**Anhydrosugars (ng m^{-3})**	165.39 ± 93.59	377.35 ± 170.59	251.66 ± 107.02	398.28 ± 116.57	645.52 ± 65.13	268.47 ± 109.78
**Levoglucosan(Levo) (ng m^{-3})**	140.02 ± 80.90	304.43 ± 126.73	216.65 ± 96.76	319.14 ± 89.77	491.50 ± 25.36	224.89 ± 92.15
**Primary sugars (ng m^{-3})**	13.10 ± 5.64	18.41 ± 10.67	9.83 ± 4.88	20.92 ± 7.93	33.70 ± 7.29	14.39 ± 7.25
**Sugar alcohols (ng m^{-3})**	7.80 ± 6.46	11.83 ± 7.94	5.90 ± 2.36	12.64 ± 5.09	24.99 ± 7.39	7.92 ± 4.73
**L/M**	14.61 ± 4.43	13.00 ± 4.21	15.46 ± 3.44	12.00 ± 4.08	9.43 ± 2.92	16.79 ± 6.50
**n-Alkanes (ng m^{-3})**	105.64 ± 39.38	153.19 ± 107.43	82.20 ± 24.91	176.72 ± 119.68	271.86 ± 71.53	171.62 ± 94.82
**CPI (C_{18}-C_{36})**	1.48 ± 0.11	1.56 ± 0.17	1.53 ± 0.18	1.58 ± 0.19	1.59 ± 0.08	1.31 ± 0.12
**WNA(%)**	19.39 ± 3.58	21.56 ± 5.31	20.59 ± 5.61	22.10 ± 5.87	22.57 ± 2.54	13.62 ± 4.31
**Fatty acids (ng m^{-3})**	159.84 ± 77.06	347.92 ± 210.80	187.98 ± 71.40	366.41 ± 122.02	712.01 ± 175.11	257.05 ± 154.14
**LMW/HMW**	0.79 ± 0.27	0.70 ± 0.24	0.87 ± 0.25	0.59 ± 0.15	0.57 ± 0.12	0.92 ± 0.48
**Fatty alcohols (ng m^{-3})**	47.19 ± 24.59	78.26 ± 46.75	42.17 ± 17.83	85.70 ± 31.74	151.26 ± 40.25	47.47 ± 22.50
**LMW/HMW**	0.033 ± 0.011	0.036 ± 0.012	0.042 ± 0.011	0.032 ± 0.014	0.032 ± 0.006	0.056 ± 0.032
**PAHs (ng m^{-3})**	4.27 ± 2.40	9.44 ± 6.08	4.98 ± 1.51	10.52 ± 4.28	17.99 ± 7.75	7.38 ± 7.59
**Flu/(Flu+Pyr)**	0.51 ± 0.02	0.51 ± 0.02	0.51 ± 0.02	0.52 ± 0.02	0.52 ± 0.02	0.52 ± 0.01
**IP/(IP+BghiP)**	0.47 ± 0.06	0.48 ± 0.05	0.47 ± 0.05	0.49 ± 0.05	0.47 ± 0.04	0.51 ± 0.04
**Hopanes (ng m^{-3})**	1.09 ± 0.57	1.72 ± 0.91	0.98 ± 0.23	1.88 ± 0.63	3.21 ± 0.66	1.74 ± 0.99
**$C_{29}\alpha\beta$/$C_{30}\alpha\beta$**	1.15 ± 0.21	1.27 ± 0.18	1.28 ± 0.18	1.25 ± 0.21	1.33 ± 0.13	1.31 ± 0.26
**$C_{30}\alpha\beta$/$C_{30}\beta\alpha$**	2.14 ± 0.64	1.32 ± 0.36	1.27 ± 0.41	1.42 ± 0.35	1.19 ± 0.21	1.46 ± 0.61
**Aromatic acids (ng m^{-3})**	13.81 ± 7.23	32.24 ± 21.70	17.35 ± 4.65	33.74 ± 15.09	66.76 ± 25.02	17.40 ± 11.24
**VA/Levo**	0.014 ± 0.004	0.013 ± 0.006	0.012 ± 0.005	0.013 ± 0.006	0.016 ± 0.009	0.008 ± 0.007
**p-HBA//Levo**	0.035 ± 0.007	0.033 ± 0.015	0.028 ± 0.009	0.033 ± 0.012	0.046 ± 0.028	0.023 ± 0.013
**Dicarboxylic acid (ng m^{-3})**	8.95 ± 3.74	24.48 ± 16.63	13.25 ± 5.11	28.35 ± 17.00	42.85 ± 15.54	17.57 ± 8.75
**M/F**	0.33 ± 0.16	0.27 ± 0.13	0.28 ± 0.08	0.29 ± 0.19	0.20 ± 0.04	0.43 ± 0.53
**Total detected organic compounds (ng m^{-3})**	527.07 ± 240.89	1054.84 ± 552.46	616.30 ± 213.44	1135.16 ± 353.41	1970.15 ± 357.69	811.00 ± 340.77
**Species/Total detected organic compounds (%)**						
**Sugar compounds**	34.21 ± 5.59	39.86 ± 5.70	42.59 ± 6.78	38.62 ± 4.05	36.23 ± 3.73	36.90 ± 7.82
**Anhydrosugars**	29.95 ± 6.12	37.01 ± 5.84	39.97 ± 6.88	35.61 ± 4.13	33.25 ± 3.70	34.05 ± 7.96
**Levoglucosan(Levo)**	25.29 ± 5.55	30.51 ± 6.20	34.16 ± 6.40	28.89 ± 4.75	25.55 ± 4.26	28.83 ± 8.15
**Primary sugars**	2.76 ± 1.14	1.76 ± 0.61	1.64 ± 0.71	1.89 ± 0.61	1.72 ± 0.24	1.87 ± 0.97
**Sugar alcohols**	1.49 ± 0.74	1.08 ± 0.31	0.98 ± 0.31	1.12 ± 0.31	1.26 ± 0.24	0.99 ± 0.37
**n-Alkanes**	21.32 ± 5.26	14.33 ± 4.73	13.89 ± 3.07	14.98 ± 6.56	13.63 ± 1.52	21.27 ± 7.38
**Fatty acids**	30.29 ± 4.40	32.09 ± 4.37	30.26 ± 3.79	32.45 ± 4.54	35.82 ± 3.06	30.37 ± 6.49
**Fatty alcohols**	8.79 ± 1.66	7.26 ± 1.85	6.94 ± 2.68	7.44 ± 1.08	7.57 ± 0.73	5.87 ± 1.60
**PAHs**	0.79 ± 0.26	0.90 ± 0.27	0.86 ± 0.29	0.93 ± 0.23	0.90 ± 0.34	0.85 ± 0.51

**Hopanes**	0.21 ± 0.08	0.17 ± 0.05	0.18 ± 0.07	0.17 ± 0.02	0.16 ± 0.00	0.22 ± 0.07
**Aromatic acids**	2.61 ± 0.56	3.02 ± 0.79	2.95 ± 0.73	2.96 ± 0.83	3.34 ± 0.93	2.17 ± 0.92
**Dicarboxylic acid**	1.77 ± 0.36	2.39 ± 1.02	2.34 ± 0.99	2.46 ± 1.03	2.33 ± 1.28	2.35 ± 1.10

LMW: low molecular weight; HMW: high molecular weight;

LMW/HMW: $(C_{18}+C_{19}+C_{20}+C_{21}+C_{22}+C_{23}+C_{24}+C_{25}+C_{26})/(C_{27}+C_{28}+C_{29}+C_{30}+C_{31}+C_{32}+C_{33}+C_{34}+C_{35}+C_{36})$ for n-alkanes;

$(C_{10:0}+C_{11:0}+C_{12:0}+C_{13:0}+C_{14:0}+C_{15:0}+C_{16:0}+C_{17:0}+C_{18:0}+C_{19:0})/(C_{20:0}+C_{21:0}+C_{22:0}+C_{23:0}+C_{24:0}+C_{25:0}+C_{26:0}+C_{27:0}+C_{28:0}+C_{29:0}+C_{30:0}+C_{31:0}+C_{32:0})$for fatty acids;

$(C_{18}+C_{19})/(C_{20}+C_{21}+C_{22}+C_{23}+C_{24}+C_{25}+C_{26}+C_{27}+C_{28}+C_{29}+C_{30}+C_{31}+C_{32})$ for fatty alcohols;

LMW PAHs: 3-ring, and 4-ring PAHs; HMW PAHs: 5-ring, and 6-ring PAHs;

CPI: Carbon Preference Index:$2\sum C_{19}\text{-}C_{35}/(\sum C_{18}\text{-}C_{34}+\sum C_{20}\text{-}C_{36})$for n-alkanes;

WNA: wax n-alkanes percentage are calculated as excess odd homologues-adjacent homologues average, and the difference from the total n-alkanes is the petroleum-derived amount. Negative values of plant wax n-alkanes were taken as zero.
